# Supplementary figures and images for: Protein and RNA ADP-ribosylation detection is influenced by sample preparation and reagents used
Source: Life Sci Alliance. 2022 Nov 11;6(1):e202201455. doi: 10.26508/lsa.202201455 (PMC9652768; doi:10.26508/lsa.202201455)

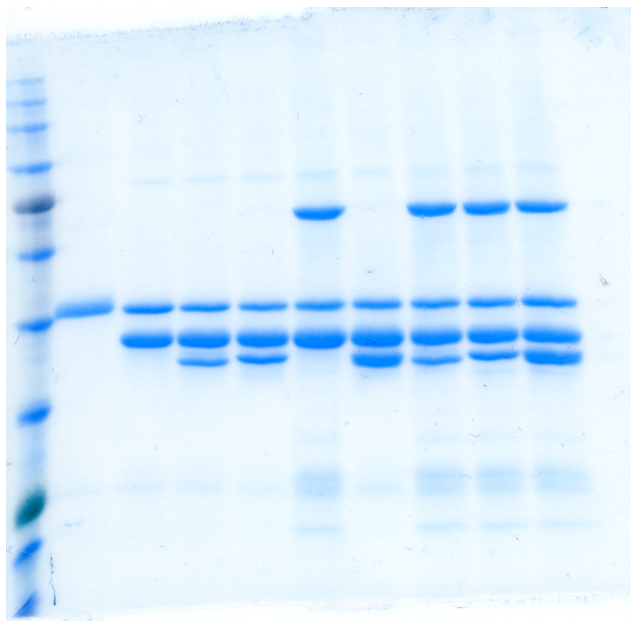

Supplement: Supplementary file 1 [file LSA-2022-01455_SdataF1.pdf]

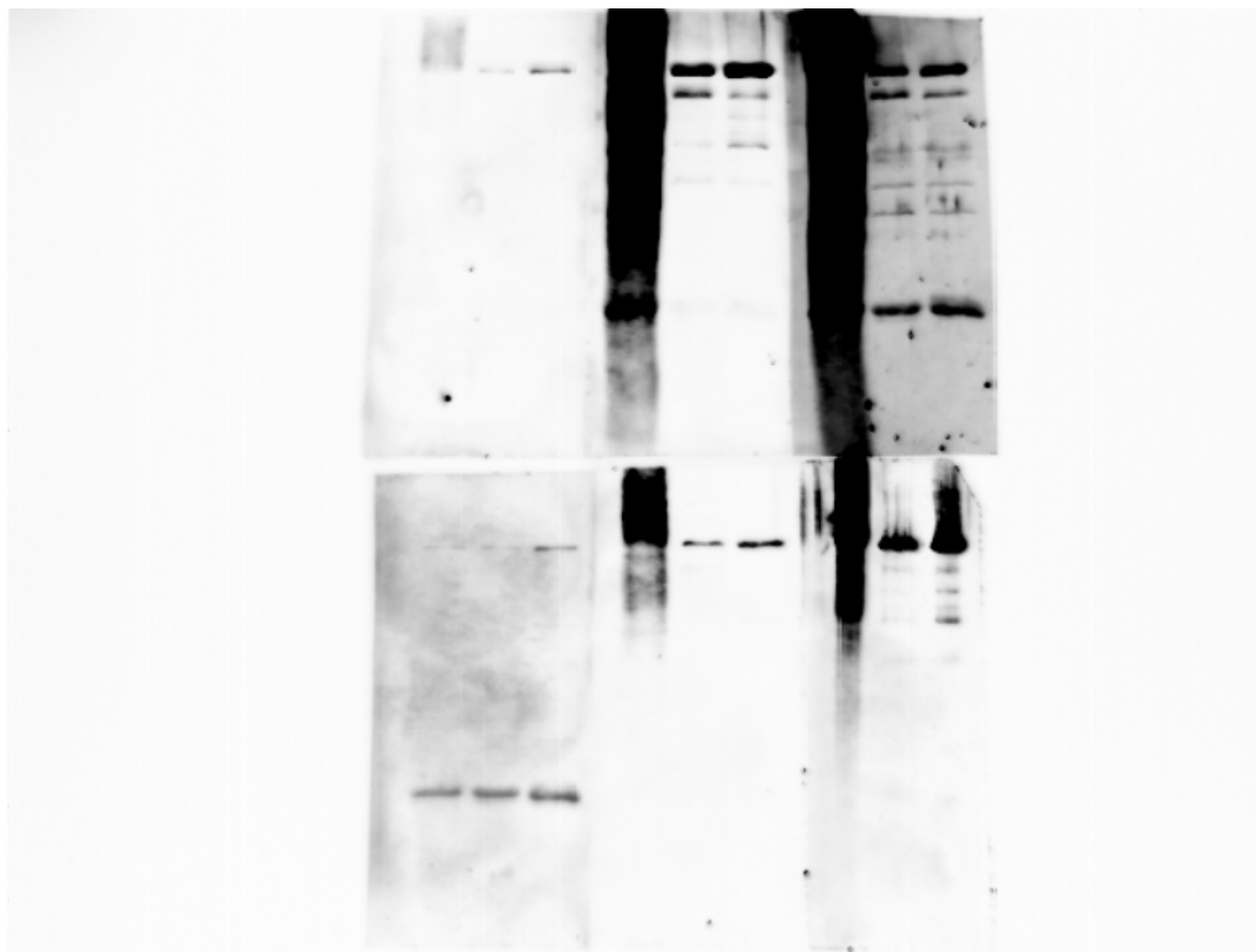

long exposure

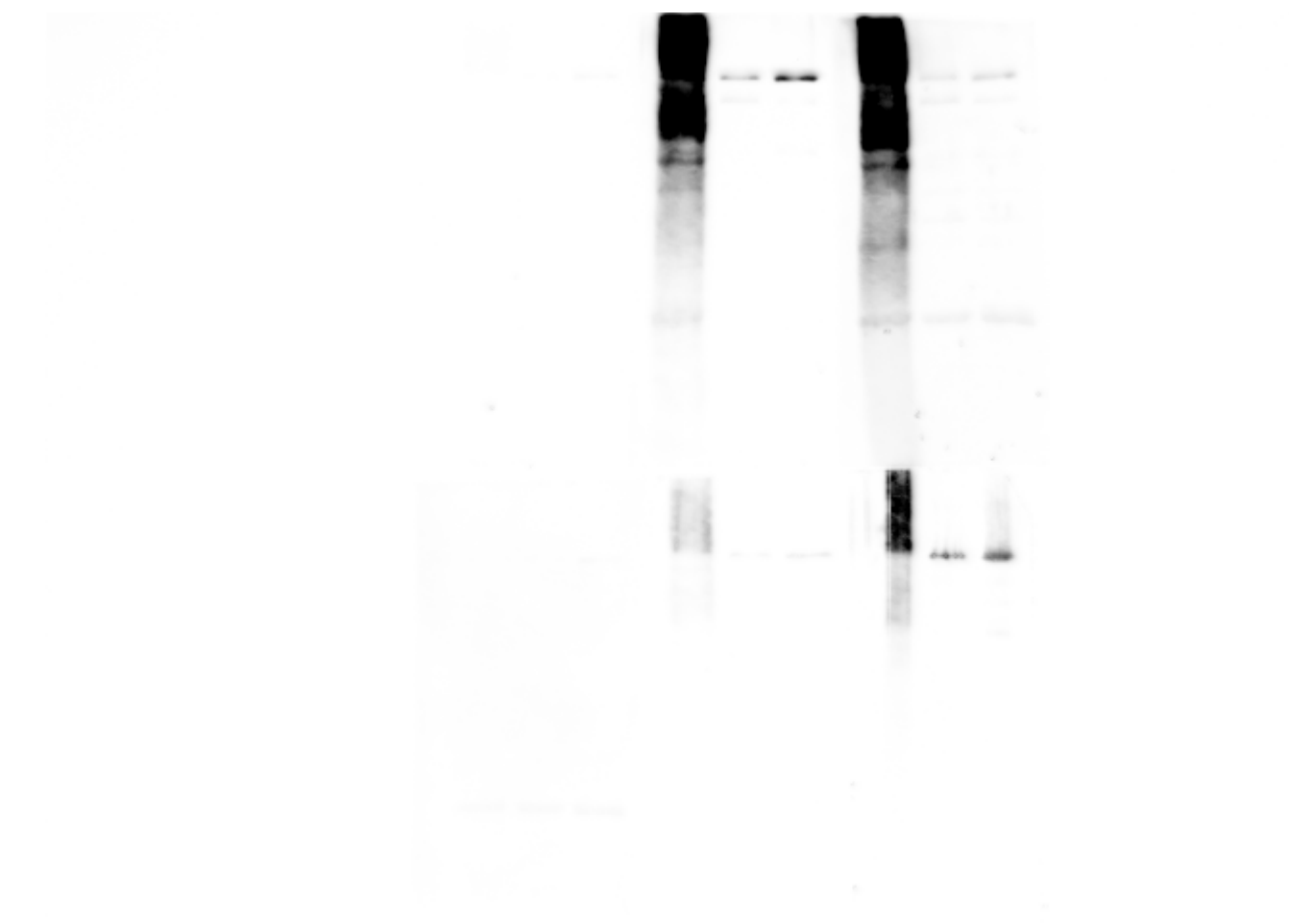

short exposure

Supplement: Supplementary file 2 [file LSA-2022-01455_SdataF2.pdf]

Marker overlay

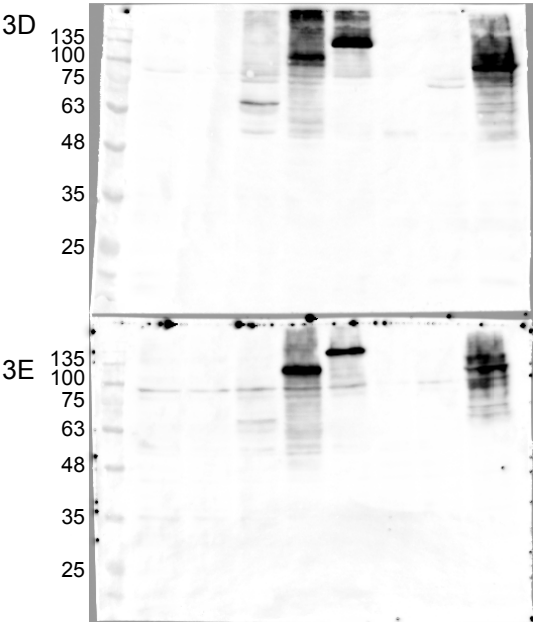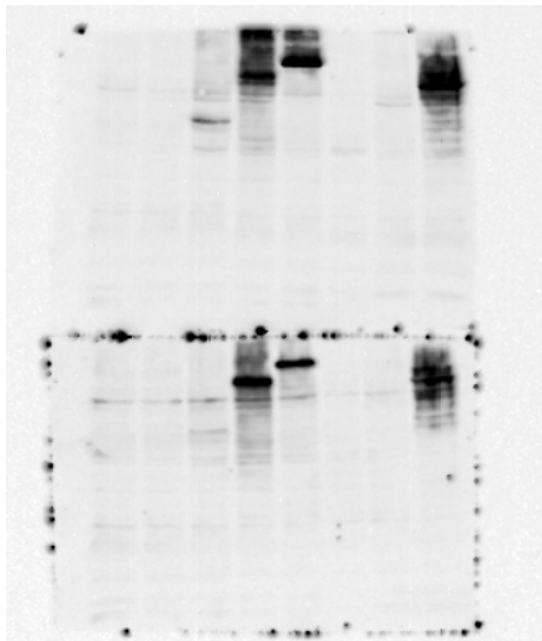

Supplement: Supplementary file 3 [file LSA-2022-01455_SdataF3.pdf]

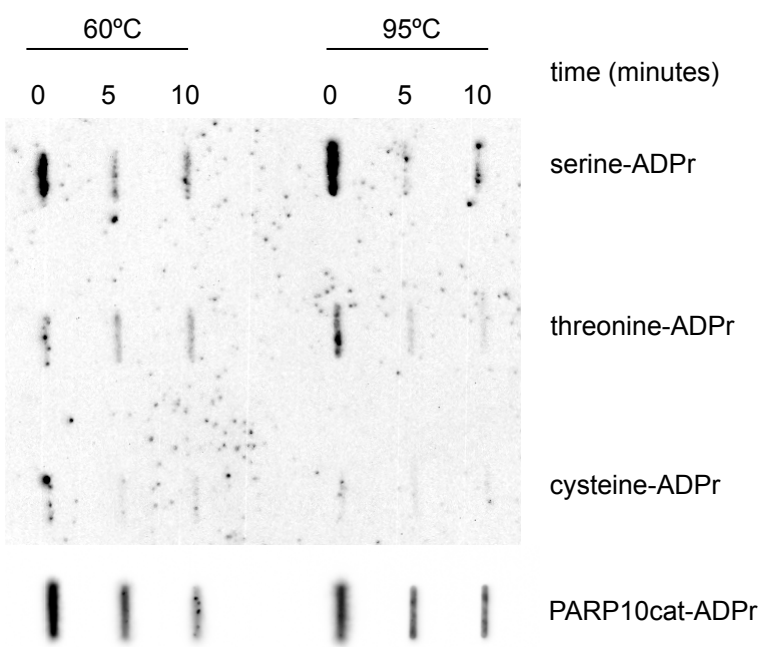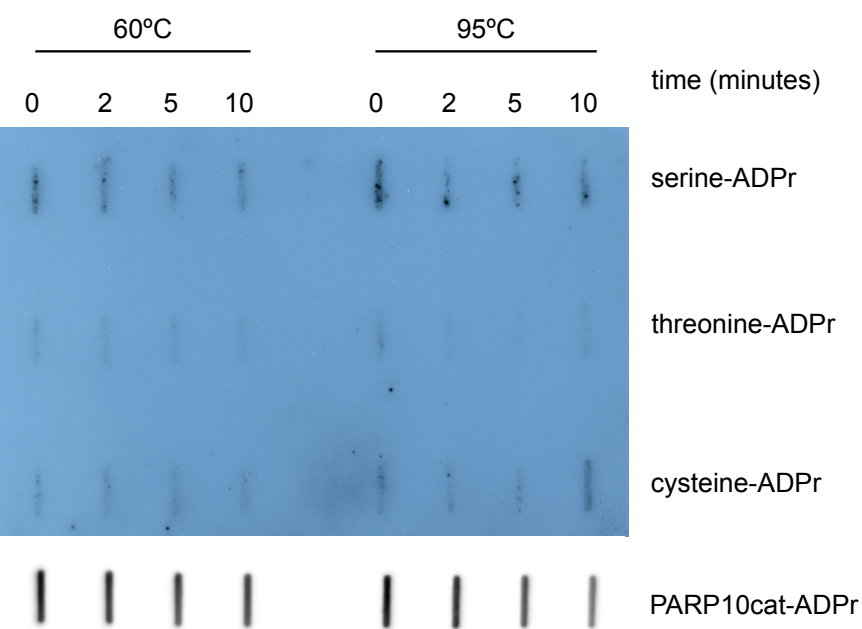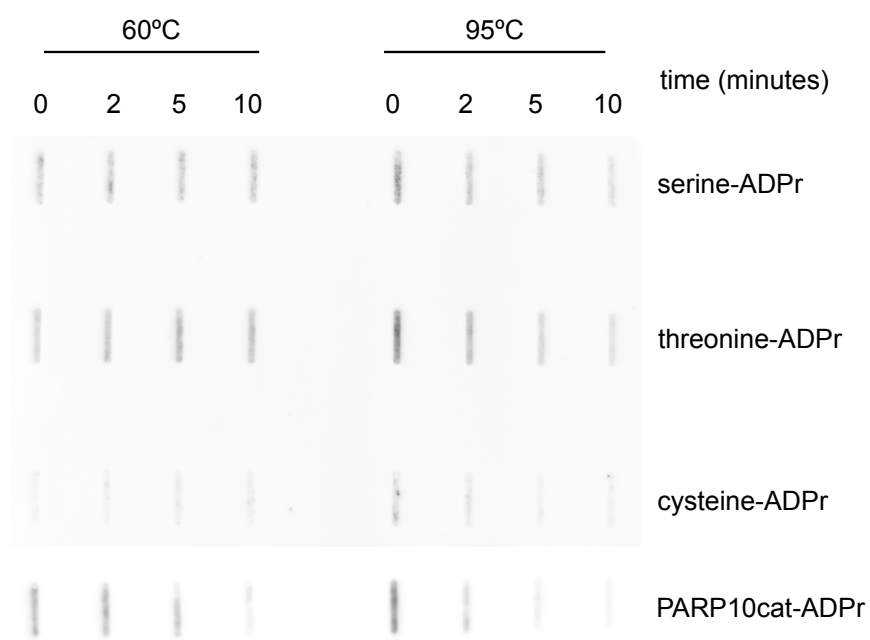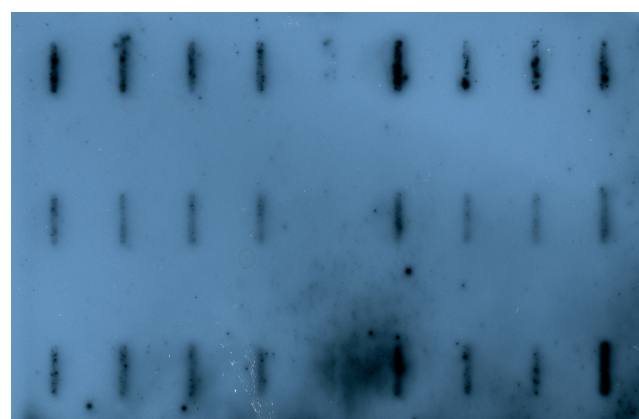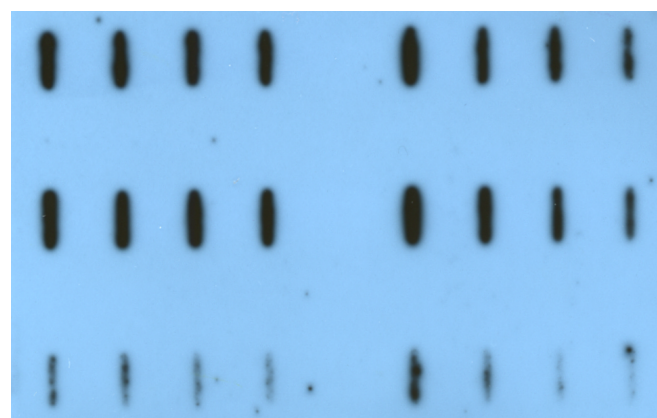

Supplement: Supplementary file 4 [file LSA-2022-01455_SdataF3_FS4.pdf]
