# Supplementary material for: Protein and RNA ADP-ribosylation detection is influenced by sample preparation and reagents used
Source: Life Sci Alliance. 2022 Nov 11;6(1):e202201455. doi: 10.26508/lsa.202201455 (PMC9652768; doi:10.26508/lsa.202201455)
Supplement: Supplementary file 5 [file LSA-2022-01455_SdataF4.pdf]

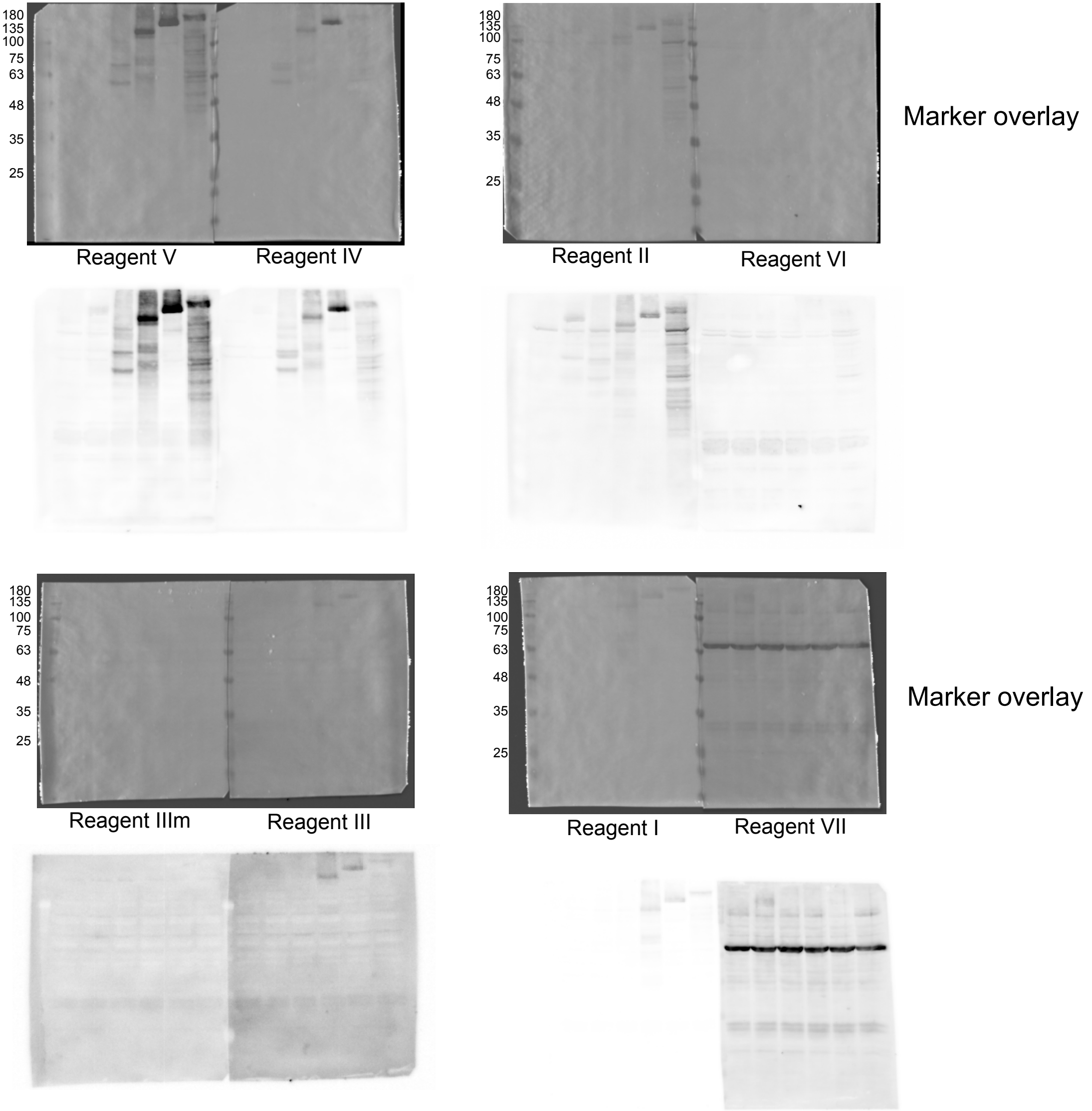

180
135
100
75
63
48
35
25
Reagent V
Reagent IV
Reagent II
Reagent VI
Marker overlay
Reagent IIIm
Reagent III
Reagent I
Reagent VII
Marker overlay
